# Optimization methods for the imputation of missing values in Educational Institutions Data

**DOI:** 10.1016/j.mex.2020.101208

**Published:** 2021-01-04

**Authors:** D. Aureli, R. Bruni, C. Daraio

**Affiliations:** a Dep. of Information Engineering, Electronics and Telecommunications, “Sapienza” University of Rome, Rome, Italy; b Dep. of Computer Control and Management Engineering, “Sapienza” University of Rome, Rome, Italy

**Keywords:** Information Reconstruction, Data imputation, Machine learning, Interconnected data, Educational Institutions

## Abstract

The imputation of missing values in the detail data of Educational Institutions is a difficult task. These data contain multivariate time series, which cannot be satisfactory imputed by many existing imputation techniques. Moreover, almost all the data of an Institution are interconnected: the number of graduates is not independent from the number of students, the expenditure is not independent from the staff, etc. In other words, each imputed value has an impact on the whole set of data of the institution. Therefore, imputation techniques for this specific case should be designed very carefully. We describe here the methods and the codes of the imputation methodology developed to impute the various patterns of missing values which appear in similar interconnected data. In particular, a first part of the proposed methodology, called ``trend smoothing imputation'', is designed to impute missing values in time series by respecting the trend and the other features of an Institution. The second part of the proposed methodology, called ``donor imputation'', is designed to impute larger chunks of missing data by using values taken form similar Institutions in order to respect again their size and trend.

• Trend smoothing imputation can handle missing subsequences in time series, and is given by a weighted combination of: (a) weighed average of the other available values of the sequence, and (b) linear regression.
• Donor imputation can handle full sequence missing in time series. It imputes the Recipient Institution using the values taken from a similar institution, called Donor, selected using optimization criteria.
• The values imputed by our techniques should respect the trend, the size and the ratios of each Institution.

## Graphical abstract

**Figure:**
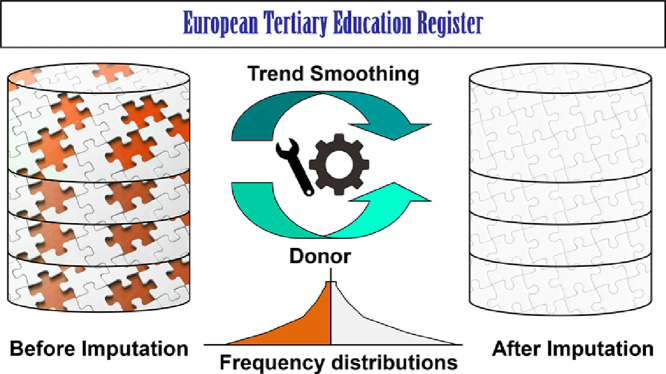

Specifications table

**Table:** 

Subject Area:	Machine Learning
More specific subject area:	Data Imputation
Method name	Trend Smoothing Imputation and Donor Imputation:
Name and reference of original method:	R. Bruni, C. Daraio, D. Aureli: Imputation Techniques for the Reconstruction of Missing Interconnected Data from Higher Educational Institutions, Knowledge-Based Systems (2020) 106512, https://doi.org/10.1016/j.knosys.2020.106512.
Resource availability:	The Data for running the codes are described in R. Bruni, C. Daraio, D. Aureli: Information Reconstruction in Educational Institutions Data from the European Tertiary Education Registry, Data in Brief (2020) Vol 34, 106611, https://doi.org/10.1016/j.dib.2020.106611.

## Introduction

Universities and other organizations providing higher level education are collectively called Higher Education Institutions (HEIs). The data describing each specific HEI, for example the number of students, the number of graduates, etc., are needed to analyze and evaluate the educational systems [1]. Unfortunately, in many cases, these data contain a substantial amount of missing values. For example, if the number of students in a given year for a given university does not appear in our dataset, but it should have been registered because that university was active and running in that year, that information is marked as a *missing value*. The presence of missing values hinders many important analyses and evaluations. Thus, the reconstruction of these missing values is often needed to work on similar data.

Imputation consists in replacing the missing values with feasible values being as similar as possible to the original values that have been lost and are now unknown [[2], [6]]. In this work we study the important case of the ETER database (European Tertiary Education Register), which contains detail data of the European HEIs, and we present methods for the imputation of its missing values. This is a difficult imputation case, because HEI data contain multivariate time series, which cannot be satisfactory imputed by many of the existing imputation techniques. Moreover, almost all the data of an Institution are interconnected: the number of graduates is not independent from the number of students, the expenditure is not independent from the staff, etc. In other words, each imputed value may impact on the situation of the whole institution. Therefore, these data are defined as "interconnected" data, and the techniques to impute them should be carefully designed.

This work describes in detail the methods and the codes implementing the methodology proposed in [3] to impute missing values in interconnected data. In particular, a first part of the proposed methodology, called ``trend smoothing imputation'', is designed to impute missing values in time series by respecting the trend and the other features of an Institution. The second part of the proposed methodology, called ``donor imputation'', is designed to impute larger chunks of missing data by using values taken form similar Institutions in order to respect again their size and trend. All the actual Python codes can be downloaded from [4].

The proposed methodology appears capable to reconstruct the information of the missing values without introducing statistically significant changes in the dataset, and the imputed values result to be close enough to the original values, as shown by experiments in [3].

## *Method details

The proposed imputation methodology is composed by two main imputation techniques: trend smoothing imputation and donor imputation. Trend smoothing imputation is designed to handle missing subsequences in time series, and the imputed value is given by a weighted combination of: (a) weighted average of the other available values of the sequence, and (b) linear regression.

Donor imputation is designed to handle full sequences missing in time series, or even full-except-one subsequences. It imputes the Recipient Institution by using the values taken from a similar institution, called Donor, selected using optimization criteria. The full mathematical details of the methodology are explained in [3]. The original ETER dataset, possibly integrated with bibliometric information, and the imputed dataset, are available in several variants from [5].

The implementation of the methods is in Python 3. To use the codes provided in this work, the user should install the open-source Anaconda Python distribution, available from www.anaconda.com. In particular, the necessary elements in Anaconda are:

• the core Python 3 language;
• Python Integrated Development Environments and Code Editors, for example Spyder, or Jupyter Notebook;
• conda, Anaconda own package management system;
• in addition to default Anaconda packages like os, math, random, etc., the following additional packages must be installed from Anaconda package repository: pandas, numpy, scikit-learn, tqdm.

Then, the user can open and run the code files, for instance in Spyder, and choose as working directory that where the code files are. The code files could also be opened in another IDE. To use Jupyter Notebook, for example, the code files should be imported with command%load <file name with full path> or using package ipynb-py-convert.

Pandas, Numpy,Scikit-Learn are the main libraries used by our methodology. They enable the data processing steps and allow the user to handle Excel file, reading them on Python Environment, analyze all the metrics with mathematical functions and finally develop the ML model for the imputation. If the user receives errors regarding these modules (e.g. ImportError: No module named module_name) they should be reinstalled by means of conda installer.

## Python codes

We provide here a user-oriented description of each of the code files, or modules, implementing the described methodology. The names of the code files are: 1_smooth_imputation.py, 2_merge_smoothfiles.py, 3_donor_imputation.py, 4_donor_imputation_relaxed.py, 5_smooth_after_donor.py, 6_merge_smoothfiles.py, 7_add_ratios_and_trends.py. All these files are contained in the compressed archives downloadable from [4].

**1_smooth_imputation.py**: this module works on the ETER dataset **original_dataset.xlsx** and performs the described trend smoothing imputation on one or more variables (students, graduates, etc.) specified by the user by means of numbers from 0 to 9 in the first part of the code. For each of those variables, it produces a file **fileout_<nameofthevariable>.xlsx** containing only two columns: **eter_id** of the institution and **imputation**, containing all the values of the treated variable after the smooth imputation, that is, original values and imputed values. Note that this column will still contain a number of missing because trend smoothing imputation is used only for subsequences of missing values being adjacent to at least two non-missing values in the same time series, while subsequences adjacent to only one non-missing value or full sequences missing are treated by donor imputation as explained below. All these files are created in a subfolder of the working directory called **output_smooth**.

**2_merge_smoothfiles.py**: this module takes all the files **fileout_<nameofthevariable>.xlsx** generated by the previous module and contained in **output_smooth**, the dataset **original_dataset.xlsx**, and produces, again in folder **output_smooth**, a file **fileout_smooth_complete.xlsx** containing all the dataset obtained after the trend smoothing imputation. This module also operates some minor postprocessing on the imputed values in order to improve their homogeneity.

**3_donor_imputation.py**: this module takes the file **fileout_smooth_complete.xlsx** from folder **output_smooth** and produces, in another subfolder of the working directory called **output_donor**, the result of the donor imputation in the file **fileout_donor.xlsx**. This file contains both original values and imputed values, and may still contain some missing values whenever a suitable donor was not available for some institution. For each of the treated variables, this file contains a column with the original value, a column with the corresponding value after trend smoothing imputation, a column with the corresponding value after donor imputation, a column with a flag explaining which type of imputation has been performed on that value, the distance of the donor if a donor has been used, the eter_id of the donor used.

**4_donor_imputation_relaxed.py**: this module takes the file **fileout_donor.xlsx** from folder **output_donor** and produces, in the same folder, file **fileout_donor_relaxed.xlsx**, with similar structure. This latter file is the result of another cycle of donor imputation with more relaxed requirements in the choice of the donor, in order to impute the institutions which remained unimputed in the first donor imputation cycle. Since values may have inferior quality during this cycle, they are flagged differently in order to distinguish them. In principle, this code could also be reused several times, by progressively relaxing the donor requirements, to maximize the number of imputations.

**5_smooth_after_donor.py**: this module performs again trend smoothing imputation in case this operation has to be performed after donor imputation. For example, if additional years are added later in the time series, and they contain missing values, this module becomes useful. This module takes **fileout_donor_relaxed.xlsx** in **output_donor** and operates on one or more variables specified by the user by means of numbers from 0 to 9 in the first part of the code. For each of those variables, it produces, in another subfolder of the working directory called **output_after_donor**, a file **fileout_<nameofthevariable>.xlsx** containing only two columns: **eter_id** of the institution and **imputation**, containing all the values of the treated variable after this new smooth imputation step.

**6_merge_smoothfiles.py**: this module is perfectly analogous to module **2_merge_smoothfiles.py**. It takes all the files **fileout_<nameofthevariable>.xlsx** generated by the previous module contained in **output_after_donor** and the dataset **fileout_donor_relaxed.xlsx** in **output_donor** and produces, in **output_donor**, a file **fileout_donor_complete.xlsx**.

**7_add_ratios_and_trends.py**: this module takes **fileout_donor_complete.xlsx** in folder **output_donor** and creates in the working directory the final file called **imputed_dataset.xlsx**. by adding in it the computation of all the significant ratios between couples of variables and all the trends of the variables. For each of the treated variables, this file contains a column with the original value, a column with the corresponding value after trend smoothing imputation, a column with the corresponding value after donor imputation, a column with a flag explaining which type of imputation has been performed on that value, the distance of the donor if a donor has been used, the eter_id of the donor used. The names of these columns may be specified by the user in file **columns_ordered.pkl**.

Note that the user may work either on a version of ETER dataset integrated with bibliometric information, or on a version of the same dataset without the bibliometric information. This choice must be specified by the user when using the first module **1_smooth_imputation.py** and is maintained by all other modules. Clearly, the final file **imputed_dataset.xlsx** will contain such bibliometric information only if the user has chosen to consider it. For this reason, we provide two alternative compressed archives: **imputation_all.zip** and **imputation_all_bibliometrics.zip**. Each of them contains all the described code files and all the corresponding data files, including the mentioned output files. The first archive does not include bibliometric information in the files, the second archive includes it.

Consider, finally, that some of the choices operated by the procedures (for example, the choice of the donor) may in some cases be done among more than one "best" solution (in the example, several donors at minimum distance). The actual choice may therefore be different in different runs of the procedure, so the final results may be different from the examples files provided in this work, however the data quality should be perfectly equivalent.

## Declaration of Competing Interest

The authors declare that they have no known competing financial interests or personal relationships that could have appeared to influence the work reported in this paper.
